# P-1445. Strategies to Promote Adult Vaccination Against Respiratory Pathogens: A Narrative Literature Review

**DOI:** 10.1093/ofid/ofaf695.1631

**Published:** 2026-01-11

**Authors:** Sarah E Williams, Kashmira Date, Jennifer Eeuwijk, Laura Sarabia, Estelle Meroc, Bradford D Gessner, Elizabeth Begier

**Affiliations:** Pfizer Vaccines, Nashville, TN; Pfizer, Inc., Atlanta, GA; P95 Clinical and Epidemiology Services, Rotterdam, Zuid-Holland, Netherlands; P95, mexico city, Distrito Federal, Mexico; P95 Pharmacovigilance and Epidemiology Services, Leuven, Belgium, Leuven, Vlaams-Brabant, Belgium; EpiVac Consulting, Anchorage, Alaska; Pfizer Vaccines, Nashville, TN

## Abstract

**Background:**

For respiratory viruses, vaccination is a cost-effective strategy to prevent disease and mitigate the economic and social burden, yet uptake of respiratory vaccinations in adults is often low. We conducted a narrative literature review (NLR) of strategies implemented to improve respiratory vaccination uptake among adults.

**Table 1.**
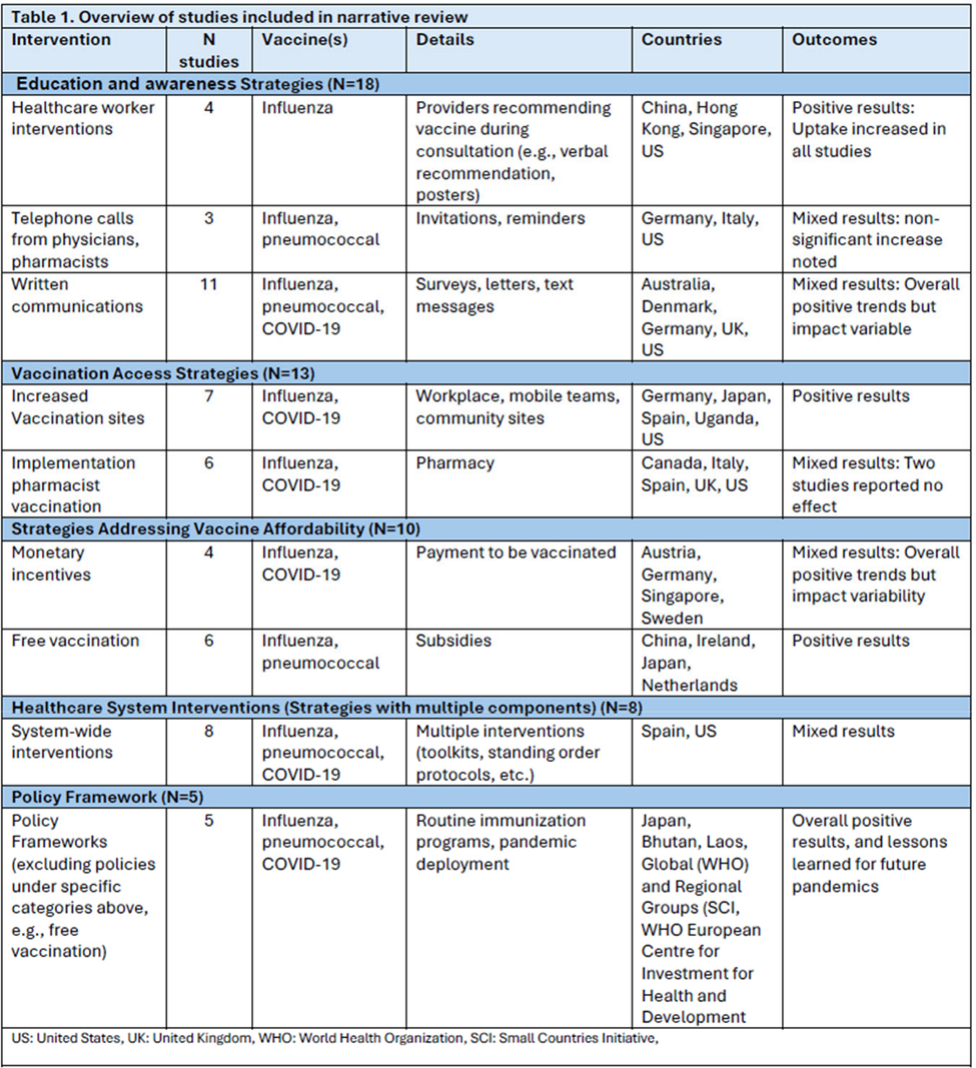
Overview of studies included in narrative review

**Table 2.**
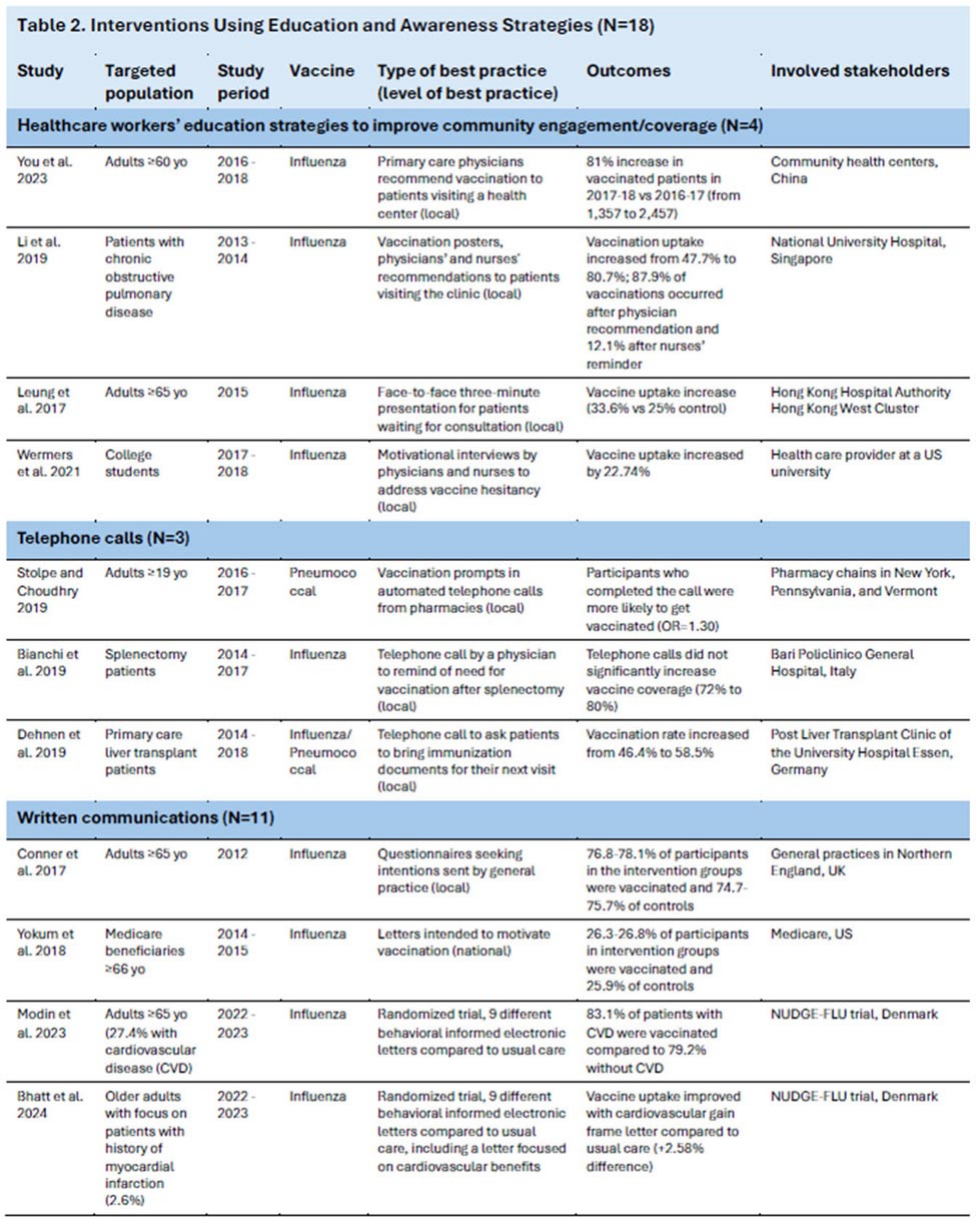
Interventions Using Education and Awareness Strategies (N=18) (Please note: First half Table 2 in this image)

**Methods:**

A search was conducted in PubMed using keywords related to vaccination strategy, programs, or implementation; respiratory diseases; and adults. Included studies were published from 2014-2024. Grey literature was identified on US Centers for Disease Control and Prevention, World Health Organization, and European Centre for Disease Prevention and Control websites. References were selected by screening titles/abstracts and full texts for eligibility, and data from the selected articles were summarized. Studies on healthcare worker (HCW) and maternal vaccination were excluded.

**Table 2.**
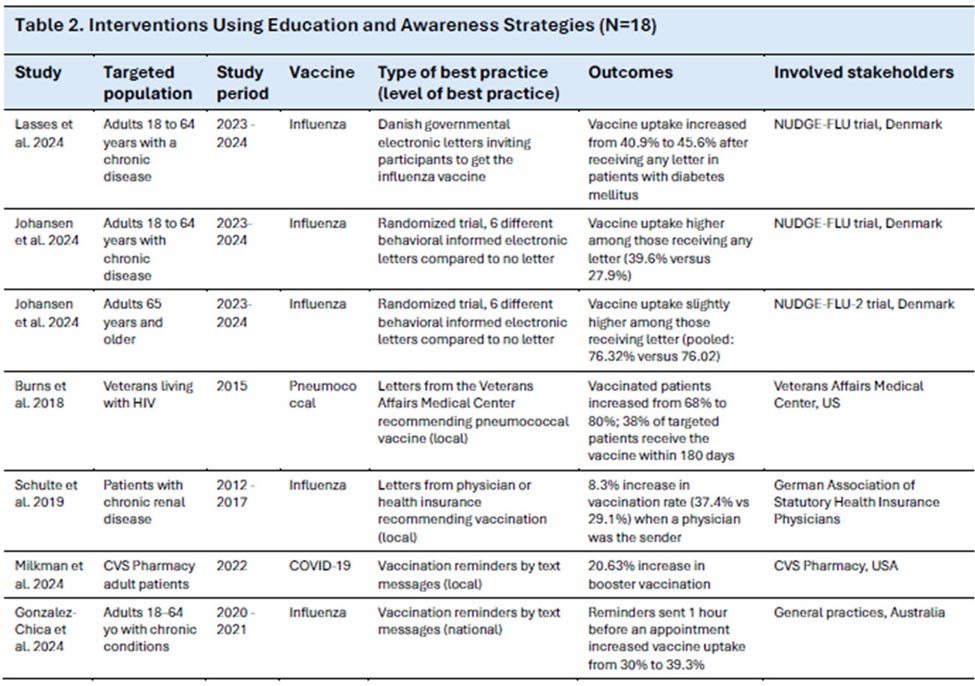
Interventions Using Education and Awareness Strategies (N=18) (Please note: Second half of Table 2 in this image)

**Results:**

Overall, 54 studies were included from 19 countries. Reported interventions included strategies to impact individual behavior, healthcare system functions, and national policy. Intervention strategy development and deployment were reported for various stakeholders (e.g., national, global, practice-level). Strategies were grouped by type of intervention (Table 1): education and awareness (N=18, Table 2), vaccination access (N=13), vaccine affordability or monetary incentives (N=10), healthcare system level interventions (N=8), and policy interventions (N=5). Interventions focused on influenza vaccination (N=31), COVID-19 vaccination (N=9), pneumococcal vaccination (N=8), or multiple adult respiratory vaccines (N=6). Impact on vaccination uptake or intent varied. Interventions including HCW (e.g., verbal recommendations), increased vaccination sites, or free vaccines consistently reported positive impacts.

**Conclusion:**

This NLR described a wide range of interventions to improve adult respiratory vaccination rates. Results highlight the importance of tailored approaches to vaccination, considering the local epidemiological context and specific barriers. The compiled examples provide a comprehensive framework for decision-makers to improve adult immunization initiatives.

**Disclosures:**

Sarah E. Williams, MD, MPH, Pfizer, Inc.: Employee|Pfizer, Inc.: Stocks/Bonds (Private Company) Kashmira Date, MD. MPH, J&J: Prior employee|Pfizer, Inc.: employee|Pfizer, Inc.: Stocks/Bonds (Private Company) Jennifer Eeuwijk, MSc, Pfizer: Grant/Research Support Laura Sarabia, PhD, Pfizer, Inc.: Contractor Estelle Meroc, DVM, MPH, PhD, Pfizer, Inc.: Contractor Bradford D. Gessner, MD, MPH, Pfizer: Stocks/Bonds (Public Company) Elizabeth Begier, MD, M.P.H., Pfizer: I am an employee.|Pfizer: Stocks/Bonds (Public Company)

